# Effective and Efficient Herbst Appliance Therapy for Skeletal Class II Malocclusion Patient with a Low Degree of Collaboration with the Orthodontic Treatment

**DOI:** 10.1155/2015/986597

**Published:** 2015-03-10

**Authors:** Bernardo Quiroga Souki, Barbra Duque Costa Bastos, Luana Fialho Ferro Araujo, Wagner Fernando Moyses-Braga, Mariele Garcia Pantuzo, Paula Loureiro Cheib

**Affiliations:** ^1^Graduate Program in Orthodontics, School of Dentistry, Pontifical Catholic University of Minas Gerais, 30535-610 Belo Horizonte, MG, Brazil; ^2^School of Dentistry, Pontifical Catholic University of Minas Gerais, 30535-610 Belo Horizonte, MG, Brazil

## Abstract

The current concept for effective and efficient treatment of skeletal Class II malocclusion prescribes that interceptive approach should be delivered during the pubertal growth stage. However, psychosocial issues and a greater risk of dental trauma are also factors that should be addressed when considering early Class II therapy. This paper reports a case of a patient that sought orthodontic treatment due to aesthetic discomfort with the incisors' protrusion. Two previous treatments failed because patient's collaboration with removable appliances was inadequate. Given his history of no collaboration and because the patient was in the prepubertal stage, it was decided to try a different approach in the third attempt of treatment. Traumatic injury protective devices were used during the prepubertal stage and followed by Herbst appliance and fixed multibrackets therapy during the pubertal stage, resulting in an adequate outcome and long-term stability.

## 1. Introduction

Class II malocclusion is highly prevalent worldwide [1–4] and its treatment is one of the most frequent in the orthodontic offices [5, 6]. When a severe skeletal disharmony is involved in the etiology of the malocclusion and patient's low collaboration is expected, a challenging situation is established to orthodontists.

This paper describes the comprehensive orthodontic treatment of a child with a severe Class II malocclusion associated with mandibular deficiency. The patient's parents reported a lack of collaboration in previous orthodontic treatment. The current concepts for efficiency and effectiveness on Class II treatment were followed and a discussion on the importance of Class II treatment timing in the search of excellence is also offered.

## 2. Case Report

**Figure 1:**
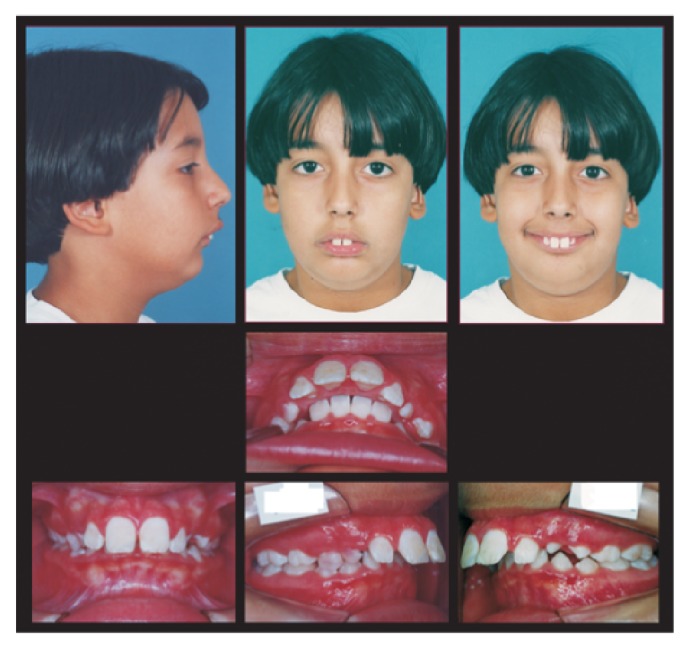
Pretreatment extraoral and intraoral photographs.

The 10-year-old boy was referred to orthodontic treatment by his pediatric dentist. The chief complaint was “the frontal teeth are too much advanced.” The patient had a marked convex and unaesthetic facial profile due to the severe mandibular deficiency. He also had a great exposure of maxillary incisors, the absence of passive lip seal, and an increased lower anterior facial height (Figure 1).

During the first consultation interview, it was reported that the patient was mouth breathing, lip trapping, and tongue thrusting. Previously two treatments with interceptive orthodontic appliances (Balters Bionator and Headgear) had been performed unsuccessfully. However, the treatments' failure in achieving an adequate outcome was associated with the lack of patient collaboration in the use of the prescribed devices.

Intraoral examination showed late mixed dentition, a complete Class II division 1 malocclusion, 15 mm of overjet, deep overbite (100%), and no dental crowding. The lower incisors impinged on the palate mucosa during occlusion (Figure 1).

**Figure 2:**
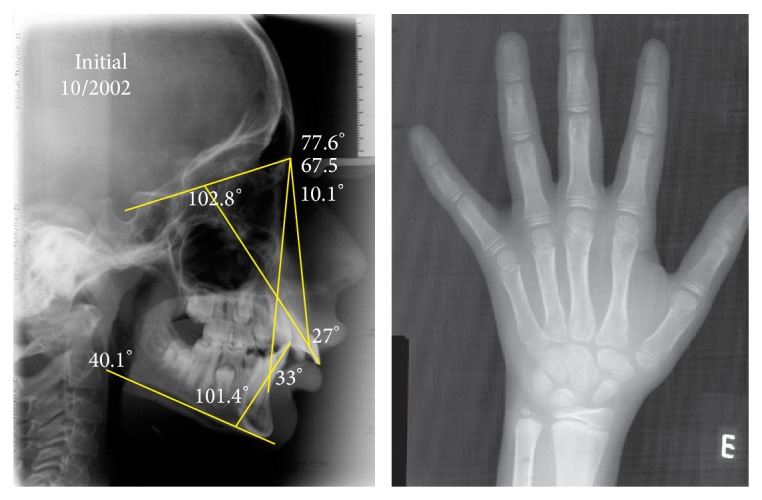
Pretreatment lateral cephalometric radiography; cephalogram; and hand-wrist radiography.

The lateral cephalometric radiograph showed a skeletal mandibular Class II relationship (SNA, 77.6°; SNB, 67.5°; ANB, 10.1°) and vertical growth pattern (SNGoGn°, 40.1°). The incisors were proclined (1.NA, 27°; 1.NB, 33°; IMPA, 101.4°). Based on the cervical vertebrae maturation method (CVM) (stage CS1) and on the hand-wrist radiographic method (HWR) (absence of sesamoid bone), the patient was prepubertal (Figure 2).

At this point, the orthodontic treatment could be carried out using a headgear or several types of functional appliances (Bionator, Twin Block, Bimler, and Frankel, e.g.). However, due to the reported lack of collaboration, an alternative treatment plan using a fixed orthopedic jumping device (Herbst appliance, HA) was presented to patient and parents.

**Figure 3:**
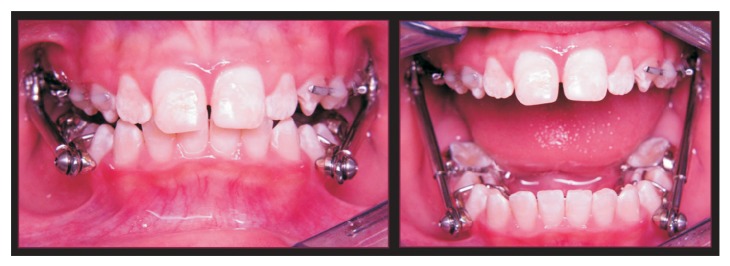
Herbst appliance immediately after insertion.

**Figure 4:**
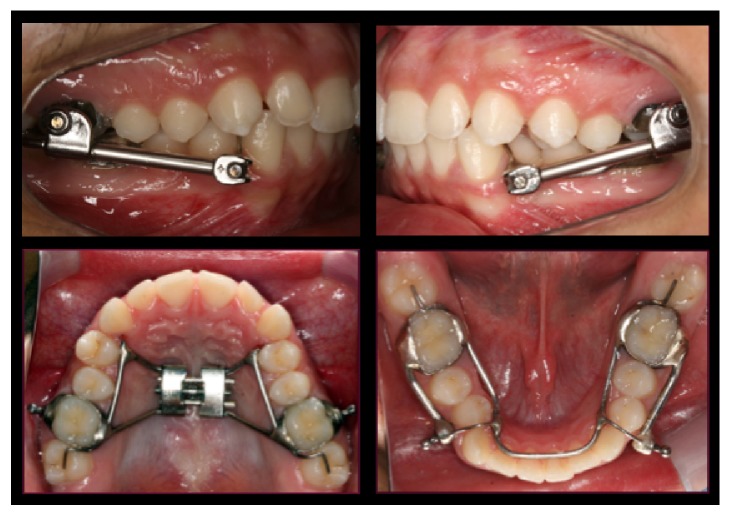
Telescopic Herbst appliance design. Hyrax expander and a heavy wire lingual arch add stability and increase the dental anchorage. Please note that this image is not from the reported case.

In order to achieve greater efficiency and effectiveness the treatment was postponed to the patient's pubertal stage of maturation. While waiting for the patient's pubertal growth spurt, traumatic injury protection devices were implemented, as a 0.40′′ plastic retainer during sports activities and a lip-bumper to avoid lower “lip trap.” When the patient reached 11 years and 4 months (CS3 stage of skeletal maturation), the HA was installed (Figure 3). The HA design included articulated bilateral telescoping arms, positioned in both maxillary and mandibular arches. The pivots were welded to a heavy cantilever wire, extending from the lower first permanent molars bands (TP Orthodontics, La Porte, IN, USA) to the cuspid region of the mandibular arch. In the maxillary arch, the pivots were welded to the first permanent molars' bands. A Hyrax expander and a 1.0 mm stainless steel lower lingual holding arch (LLHA) were added to the HA structure to improve appliance stability and transversal relationship. Excessive mandibular incisors proclination was reduced with the heavy wire LLHA.  Figure 4 shows images from another patient with the same HA design used in the current case.

**Figure 5:**
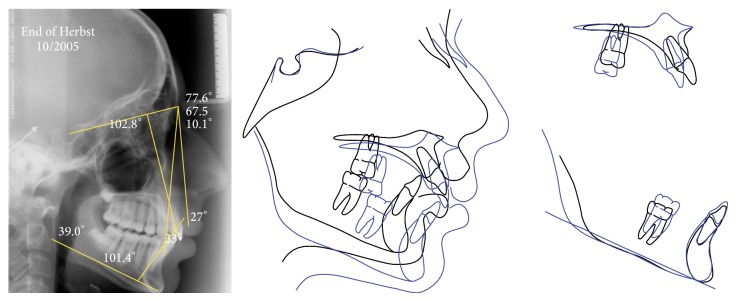
Lateral cephalometric radiography at the end of Herbst appliance phase, and superimposition tracings between pretreatment and the end of Herbst appliance phase.

**Figure 6:**
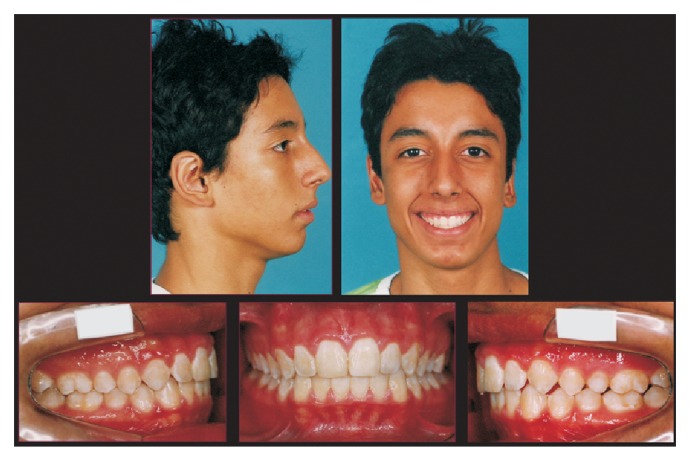
Posttreatment extraoral and intraoral photographs.

**Figure 7:**
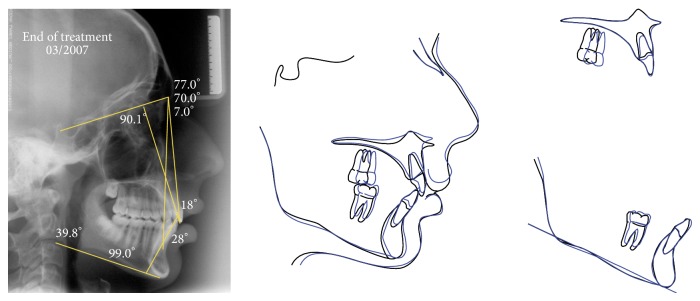
Lateral cephalometric radiography at the end of treatment, and superimposition tracings between the end of the Herbst appliance phase and fixed appliance phase.

The simultaneous occurrence of excessive overjet, severe skeletal discrepancy, and the ongoing pubertal growth maturation was the determining factor on the decision for choosing two phases of sagittal activation. Each one comprised of 8 months of HA. The HA was removed for 4 months between the two active activations to allow the patient a treatment break. The cephalometric superimposition displayed significant mandibular growth during HA therapy. By this time, the patient was finishing his pubertal stage (CS4) (Figure 5). To achieve a better dental intercuspation, a 17-month treatment with edgewise multibrackets, immediately following HA, was necessary. For every new alignment and leveling wire, the patient was requested to use 1 week of Class III elastics (3/8), thus reducing the lower incisor's proclination. The end of the treatment occurred in the postpubertal stage (CS5) (Figures 6 and 7), which might have contributed to the long-term occlusal stability.

From aesthetical and myofunctional perspectives, the treatment achieved good results, improving the initial profile, decreasing the incisors exposure during rest, and reaching a passive lip seal (Figure 6). The maxillary incisors positioning in the basal bone also showed an improvement (1.NA, 17°), while the mandibular incisors maintained their original position (1.NB, 28°; IMPA, 104°) providing an adequate overbite (3.0 mm) and overjet (2.0 mm). The treatment contributed to an increase in the mandibular prognathism (SNB, 70°), better maxillary positioning (SNA, 77°), and an improvement in sagittal relationship between maxilla and mandible (ANB, 7.0°). The skeletal pattern of vertical growth showed no clinically significant changes (SNGoGn, 39°) (Figure 7).

**Figure 8:**
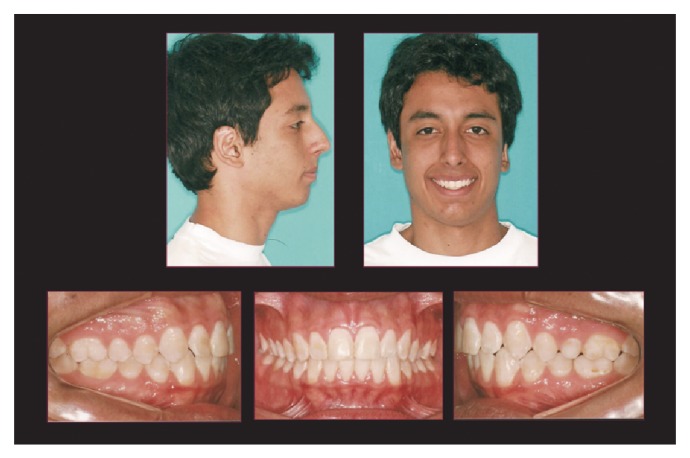
5-year postretention photographs.

**Figure 9:**
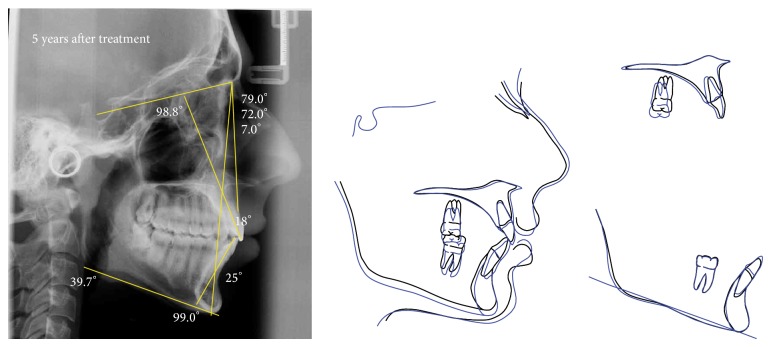
Lateral cephalometric radiography at 5 years after retention, and superimposition tracings between the end of fixed appliance phase and 5 years after retention.

After braces removal, the patient was instructed to a night use of removable plastics retainers. Five years after debonding, great stability and no relapses were observed. The patient reported no use of the removable retainers since brackets were removed (Figures 8 and 9).

## 3. Discussion

Current evidence-based guideline on the treatment timing of Class II malocclusion defines that skeletal maturation, psychosocial aspects, and the risk of traumatic injuries must be considered [7]. Randomized clinical trials, in the past decade, concluded that in the search for an effective and efficient outcome, severe Class II patterns should be approached in one-phase treatment, including the pubertal stage of maturation [8, 9]. Treatments starting too early will extend throughout a long period and will present greater chance of relapse, besides exhausting the patient's collaboration [9, 10]. However, postponing the interceptive approach may expose the patient to bullying and to an increased risk of incisor's traumatic injury [7].

The treatments of malocclusions that rely on the patient's collaboration are less likely to achieve good results. Several aspects are associated with the patient's compliance, as the age and gender of the patient [11], the child's self-esteem [12], the patient/orthodontist relationship [13], and the duration of treatment [14]. But the type of appliance plays an important role in the final treatment outcome. Removable appliances are contraindicated when patient's compliance is a problem. In the last 30 years the HA has been widely used in orthodontics to correct Class II malocclusion associated with mandibular deficiency [14–16]. A major advantage of this fixed appliance is the independence from the patient's collaboration [15].

We believe that the successful outcome of the presented case report might be associated with three aspects: (1) an orthopedic therapy performed during the pubertal period; (2) the end of the treatment that occurred after the completion of the pubertal maturation; and (3) the use of fixed appliances, excluding the patient collaboration.

Several evaluative methods have been proposed to assess the biological age [10, 17, 18], but certainly the HWM and, in the last decade, the CVM are the most used in orthodontics. We used both HWM and CVM to the establishment of the optimum window for the orthopedic approach of this severe Class II malocclusion. Including the pubertal stage of development certainly contributed to the effective and efficient outcome and favored a greater mandibular growth [8]. However, we must call attention to the fact that excessive overjet is a risk factor to traumatic injuries in Class II division 1 young subjects. Therefore, the decision to postpone the skeletal discrepancy therapy should be synchronized with some protective devices to avoid traumatic injuries on the maxillary incisors. During the 1 year 5 months waiting period, the patient was recommended to use a 0.40′′ plastic retainer during his sports practice. He also received a full time lip bumper to eliminate “lip trap,” providing a psychosocial comfort and the reduction of the risk of trauma in the maxillary incisors.

The literature has showed that Class II malocclusion subjects present the mandibular growth pattern similar to Class I peers during the prepubertal and the postpubertal periods. However, during the pubertal growth stage, Class II adolescents have a significant smaller mandibular length (Co-Gn) gain than Class I subjects [19, 20]. Therefore, in order to avoid skeletal relapse, it is mandatory to wait for the end of the pubertal growth spurt to finish the orthopedic growth control. We ended the active orthodontic treatment at a postpubertal stage (CS5). The facial growth that the patient presented after braces removal was more balanced and did not contribute to a Class II relapse.

Patient's complaint from the tremendous discomfort immediately after HA installation is very common. But a natural adjustment will follow, and an increase in the treatment adherence after the first week is expected. In the present case, as the patient's self-esteem greatly improved after the appliance installation, it may have contributed to the collaboration to therapy. There was no breakage of the device or emergency visits during treatment, which are one of the complicating factors in this type of treatment [21].

## 4. Conclusion

A comprehensive treatment plan, not only including the concepts of effectiveness and efficiency, but also considering the psychosocial and traumatic injury risks, should be addressed when a skeletal Class II malocclusion is diagnosed. HA therapy is an alternative way when the expected patient collaboration is low.
